# Association of Hydrophobic Carboxyl-Terminal Dendrimers with Lymph Node-Resident Lymphocytes

**DOI:** 10.3390/polym12071474

**Published:** 2020-06-30

**Authors:** Yutaka Nishimoto, Misaki Nishio, Shu Nagashima, Kohei Nakajima, Takayuki Ohira, Shinya Nakai, Ikuhiko Nakase, Kei Higashikawa, Yuji Kuge, Akikazu Matsumoto, Mikako Ogawa, Chie Kojima

**Affiliations:** 1 Department of Applied Chemistry, Graduate School of Engineering, Osaka Prefecture University, 1-1 Gakuen-cho, Naka-ku, Sakai, Osaka 599-8531, Japan; db105013@edu.osakafu-u.ac.jp (Y.N.); szb02092@edu.osakafu-u.ac.jp (M.N.); sxb02093@edu.osakafu-u.ac.jp (S.N.); matsumoto@chem.osakafu-u.ac.jp (A.M.); 2 Laboratory of Bioanalysis and Molecular Imaging, Graduate School of Pharmaceutical Sciences, Hokkaido University, Kita-12 Nishi-6, Kita-ku, Sapporo, Hokkaido 060-0812, Japan; ko-nakajima@eis.hokudai.ac.jp (K.N.); a-gentilis319@eis.hokudai.ac.jp (T.O.); mogawa@pharm.hokudai.ac.jp (M.O.); 3 Department of Biological Science, Graduate School of Science, Osaka Prefecture University, 1-2, Gakuen-cho, Naka-ku, Sakai, Osaka 599-8570, Japan; szc04075@edu.osakafu-u.ac.jp (S.N.); i-nakase@21c.osakafu-u.ac.jp (I.N.); 4 Central Institutes of Isotope Science, Hokkaido University, Kita 15 Nishi 7, Kita-ku, Sapporo, Hokkaido 060-0815, Japan; higashikawa@ric.hokudai.ac.jp (K.H.); kuge@ric.hokudai.ac.jp (Y.K.)

**Keywords:** dendrimer, carboxyl terminal, hydrophobicity, lymph node, T cells, phenylalanine

## Abstract

Delivery systems to lymph node-resident T cells around tumor tissues are essential for cancer immunotherapy, in order to boost the immune responses. We previously reported that anionic dendrimers, such as carboxyl-, sulfonyl-, and phosphate-terminal dendrimers, were efficiently accumulated in lymph nodes via the intradermal injection. Depending on the terminal structure, their cell association properties were different, and the carboxyl-terminal dendrimers did not associate with any immune cells majorly. In this study, we investigated the delivery of carboxyl-terminal dendrimers with different hydrophobicity to lymph node-resident lymphocytes. Four types of carboxyl-terminal dendrimers—succinylated (C) and 2-carboxy-cyclohexanoylated (Chex) dendrimers with and without phenylalanine (Phe)—were synthesized and named C-den, C-Phe-den, Chex-den, and Chex-Phe-den, respectively. Chex-Phe-den was well associated with lymphocytes, but others were not. Chex-Phe-den, intradermally injected at the footpads of mice, was accumulated in the lymph node, and was highly associated with the lymphocytes, including T cells. Our results suggest that Chex-Phe-den has the potential for delivery to the lymph node-resident T cells, without any specific T cell-targeted ligands.

## 1. Introduction

Cancer immunotherapy aims to boost patients’ immune systems in order to kill tumor cells, which can generate a coordinated and proliferative anti-tumor response. It is applicable in various cancer types, but the efficacy remains to be improved [1]. The sentinel lymph node (SLN) is the first organ that drains tumor cells from malignant carcinomas, which is the first site of tumor-specific T cell activation [2,3]. SLN has large amounts of antigen-presenting cells and tumor-specific T cells, so great efforts are being made to explore the strategy of targeting to the SLN, as well as the SLN-resident T cells. However, concentrations of drug or antigen among cytotoxic T cells in the SLN are inappropriate, which is a cause of the limited efficacy [4]. Schmid et al. have developed CD8-conjugated poly(lactic-co-glycolic acid) (PLGA) nanoparticles (NPs) for T cell-targeting [5]. The anti-CD8a F(ab′)_2_ fragments-conjugated PLGA NPs were bound to the surface of CD8+ T cells, and these NPs were internalized into the cells. However, the efficiency of the accumulation in the lymph node and the cell internalization was low [5]. Hence, new methods for delivery to efficient lymph node-resident T cells are required.

**Figure 1:**
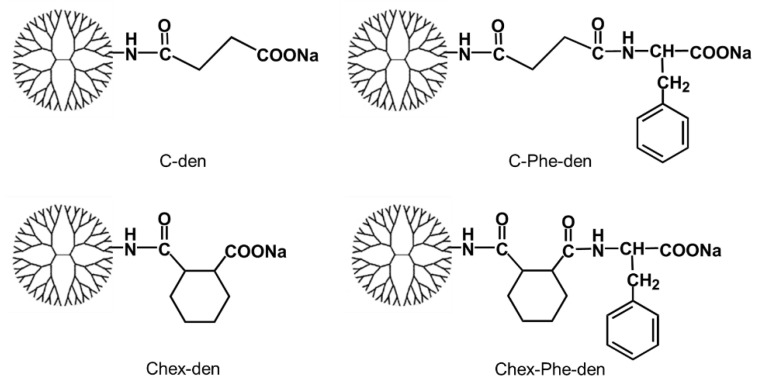
Structure of four carboxyl-terminal dendrimers with different hydrophobicity.

Dendrimers are synthetic highly branched macromolecules that are synthesized by stepwise methods and characterized by multivalent surfaces. Dendrimers have distinct capabilities to conjugate some bioactive compounds at the periphery, as well as to encapsulate them within the interior. Thus, there are many reports that show these abilities as potent nanoplatforms for drug delivery systems and imaging [6,7,8]. Several groups have reported the delivery of dendrimers to the lymph nodes [9,10,11,12,13,14]. A systematic study was carried out by using various dendrimers with different sizes and terminal groups, to optimize the dendrimers’ structures for the delivery to SLN. Anionic carboxyl-terminal dendrimers of greater than generation-4 (G4) were accumulated in the lymph node by intradermal administration, but cationic amino- and nonionic acetyl-terminal dendrimers were not [9]. The accumulation in the lymph node and their association with lymph node-resident immune cells of different anionic dendrimers, such as carboxyl-, sulfonyl-, and phosphate-terminal dendrimers, were then investigated. The phosphate-terminal dendrimer was recognized by the lymph node-resident immune cells expect for T cells, while the carboxyl- and sulfonyl-terminal dendrimers were not [10]. Hence, we attempted to develop a dendrimer-based delivery system into lymph node-resident T cells. Previously, Moyano et al. demonstrated a quantitative correlation between hydrophobicity of gold NPs and immune system activation. They also showed the increased immune response of cyclohexyl compounds-modified gold NPs [15]. Shima et al. reported that polymers with carboxyl groups and hydrophobic amino acids, such as phenylalanine (Phe), effectively induced the immune responses by increasing the association with dendritic cells [16]. Previously, we synthesized Phe-modified carboxyl-terminal dendrimers as a pH- and thermo-sensitive dendrimer [17]. These reports inspired us to apply hydrophobic carboxyl-terminal dendrimers for delivery into lymph node-resident lymphocytes. In this study, we examined the association with immune cells, as well as the lymph node-targeted delivery of four carboxyl-terminal dendrimers with different hydrophobicity: succinylated (C) and 2-carboxy-cyclohexanoylated (Chex) dendrimers with and without Phe (Figure 1). The association of these dendrimers with lymphocytes was examined by flow cytometry, to understand the correlation of the dendrimer structure to the association with lymphocytes. Then, Chex-Phe-den was intradermally injected at the footpads of mice. The biodistribution, the accumulation in the lymph node, and the association of these dendrimers with lymph node-resident lymphocytes were examined.

## 2. Materials and Methods

### 2.1. Materials

Amino-terminal ethylenediamine core polyamidoamine (PAMAM) dendrimers of generation-4 and generation-5 (G4 and G5) were purchased from Sigma–Aldrich Co. (St. Louis, MO, USA). 4-(4,6-dimethoxy-1,3,5-triazin-2-yl)-4-methylmorpholinium chloride (DMT-MM) was purchased from FUJIFILM Wako Pure Chemical Co. (Tokyo, Japan). *N*-(*tert*-butoxycarbonyl)-1,2-diaminoethane (Boc-ethylenediamine) and fluorescein-4-isothiocyanate (FITC) were purchased from Tokyo Chemical Industry Co., Ltd (Tokyo, Japan). 2-(4-isothiocyanatobenzyl)-diethylenetriamine pentaacetic acid (*p*-SCN-Bn-DTPA) was purchased from Macrocyclics (Plano, TX, USA). ^111^InCl_3_ and HiLyte™ Fluor 488 acid, SE (HF488) were obtained from Nihon Medi-Physics Co., Ltd. (Tokyo, Japan) and AnaSpec Inc. (Fremont, CA, USA), respectively. Phycoerythrin (PE)-conjugated antibodies, such as CD3-PE (clone REA641) and CD45R (B220)-PE (Clone REA755), were purchased from Miltenyi Biotec, Ltd. (Bergisch Gladbach, Germany).

### 2.2. Synthesis of Carboxyl-Terminal Dendrimers

C-den, C-Phe-den, Chex-den, and Chex-Phe-den of G4 and G5 were synthesized, in accordance with our previous report [17]. Each carboxyl-terminal dendrimer (21–52 mg), Boc-ethylenediamine (four equivalents to dendrimer), and DMT-MM (six equivalents to dendrimer) were dissolved in 1 mL of water and stirred overnight at room temperature. The mixture was purified by ultrafiltration, using Amicon® Ultra-4 (MWCO 3kDa, Merck Millipore Ltd., Ireland) eluting with 125 mM NaHCO_3_ aqueous solution and the subsequent deionized water. Then, lyophilization was performed to obtain Boc-ethylenediamine-modified dendrimers. The bound number of Boc-ethylenediamine to each dendrimer was estimated as 2–5 from the ^1^H NMR spectra. Then, each Boc-ethylenediamine-modified dendrimer was dissolved in 4 mL of trifluoroacetic acid (TFA) and stirred for 3 h on ice. After the evaporation, water was added and then co-evaporated several times. After the lyophilization, ethylenediamine-modified dendrimers were obtained. Then, FITC, HF488, or *p*-SCN-Bn–DTPA was reacted at the ethylenediamine termini, as follows. For labeling with FITC, each ethylenediamine-modified dendrimer of G4 (14–22 mg) was dissolved in 1 mL of dimethylsulfoxide (DMSO). Four equivalents of FITC and six equivalents of triethylamine were added to the dendrimer solution and stirred overnight at room temperature. The dendrimers were purified by dialysis (MWCO 2kDa) in DMSO. Then, lyophilization was performed, to obtain the FITC-labeled dendrimers. The bound number of FITC to each dendrimer was estimated as 2–5 from the absorbance at 495 nm of the dye-conjugated dendrimers. For labeling with HF488, ethylenediamine-modified Chex-Phe-den of G5 (20 mg, 0.3 μmol) was dissolved in 1 mL of 125 mM NaHCO_3_ aqueous solution. Additionally, four equivalents of HF488 was dissolved in 1 mL of DMSO and mixed with the dendrimer solution, and then stirred for 24 h at room temperature. For labeling with DTPA, ethylenediamine-modified Chex-Phe-den of G5 (12 mg, 0.2 μmol) was dissolved in 0.9 mL of 125 mM NaHCO_3_ aqueous solution. Seven equivalents of *p*-SCN-Bn–DTPA were added to the dendrimer solution and stirred for 24 h at room temperature. These reaction mixtures were purified by ultrafiltration, using Amicon® Ultra-4 (MWCO 3kDa) eluting with the 125 mM NaHCO_3_ aqueous solution and the subsequent deionized water. Then, lyophilization was performed to obtain HF488- and DTPA-modified Chex-Phe-den (9 mg and 5 mg, respectively). Two HF488 or two DTPA were conjugated to the dendrimer, which were estimated from the absorbance of HF488 and DTPA, in accordance with our previous report [10]. Then, DTPA-modified Chex-Phe-den was radiolabeled by incubating with ^111^InCl_3_, in accordance with our previous report [10]. 

### 2.3. Characterization

The ^1^H NMR spectra were recorded in D_2_O including NaOD using JEOL ECS-400 and ESX-400 spectrometers. The dendrimer solutions (1 mg/mL, phosphate-buffered saline (PBS)) were prepared and filtered (0.45 μm), and the ζ-potential analysis was carried out using the ELSZ-DN2 (Otsuka Electronics Co, Ltd., Osaka, Japan).

### 2.4. Animals

BALB/c mice (7–10 weeks old, female) were used in this study. Animal care, experiments, and euthanasia were approved by the Animal Care and Use Committees of Hokkaido University (15-0119) and Osaka Prefecture University (30-186 and 19-6) and carried out according to the guidelines of the committees.

### 2.5. Biodistribution

Biodistribution study was performed by the same method as our previous report [10]. Briefly, radiolabeled Chex-Phe-den (30 μL, 111 kBq) was intradermally injected into the right rear footpads of the mice, followed by massages for 0.5 min. Each mouse was sacrificed at 3 or 24 h post-injection. Blood, popliteal lymph node, heart, lung, liver, spleen, kidney, and the injection site were then collected and weighed. The radioactivity was measured using an auto well gamma counter (Automatic Gamma Counter, 2480 WIZARD 2, PerkinElmer Inc., Waltham, MA, USA). As a comparison, the results of C-den obtained in our previous report were shown in this study [10].

### 2.6. Flow Cytometry

Flow cytometry study of lymph node-resident lymphocytes was performed by the same method as our previous report [10]. Briefly, HF488-labeled Chex-Phe-den (3 nmol, 30 μL) was intradermally injected into the front and rear footpads of the mice, followed by massages for 0.5 min. Each mouse was sacrificed at 3 h post-injection, and then the popliteal and axillary lymph nodes were collected. The immune cells were obtained from these lymph nodes, after enzymatic digestion with collagenase. Then, the cells were suspended in RPMI-1640 and incubated at 1 × 10^6^ cells stained with each PE-conjugated monoclonal antibody (CD3-PE or CD45R-PE), in accordance with the manufacturer’s protocol. The stained cells were evaluated via flow cytometry, using BD FACS Calibur (BD Biosciences, Franklin Lakes, NJ, USA). As a comparison, the results of C-den obtained in our previous report were shown in this study [10].

For a comparison of four kinds of dendrimers in the cell association, BALB/c mice were perfused by PBS containing heparin under anesthesia, and their spleens were collected. The immune cells were obtained after enzymatic digestion with collagenase for 30 min. Then, 2 × 10^5^ cells were suspended in RPMI-1640 and incubated with each FITC-labeled dendrimer (5 μM) for 3 h at 37 °C or 4 °C. Then, the cells were washed and stained with each PE-conjugated monoclonal antibody (CD3-PE and CD45R-PE), as described above. The stained and non-stained cells were evaluated via flow cytometry, using GUAVA Incyte, (Luminex, Tokyo, Japan), and the mean fluorescence intensity of cells without staining with any PE-labeled antibodies, and the number of double-positive cells were analyzed.

### 2.7. Fluorescence Imaging

Fluorescence imaging study was performed by the same method as our previous report [10]. Briefly, HF488-labeled Chex-Phe-den (3 nmol, 30 μL) was intradermally injected into the right rear footpad of the mice, followed by massages for 0.5 min. Each mouse was sacrificed and skinned at 4 or 24 h post-injection. Fluorescence imaging was performed with a FluorVivo 300 small animal fluorescence imaging system (INDEC Biosystems, Santa Clara, CA, USA) equipped with a 452–487 nm excitation filter and a 500-nm long-pass emission filter. As a comparison, the results of C-den obtained in our previous report were shown in this study [10]. Image analysis was conducted with ImageJ software ver. 1.49 (http://rsb.info.nih.gov/ij/).

## 3. Results

### 3.1. Association of Hydrophobic Carboxyl-terminal Dendrimers with Immune Cells

**Figure 2:**
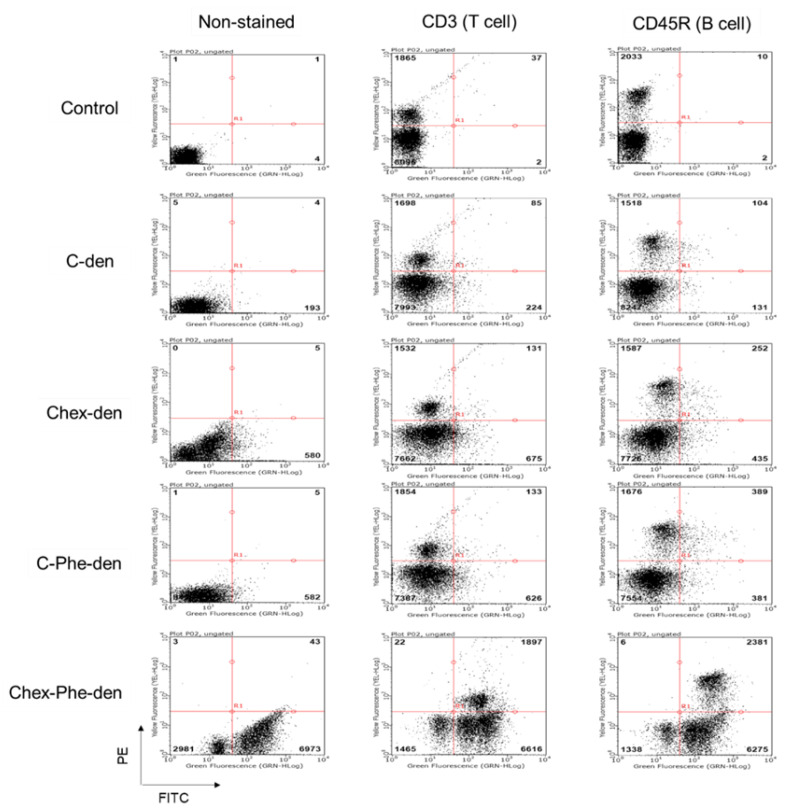
Flow cytometry of non-stained and Phycoerythrin (PE)-stained immune cells, treated with the green fluorescent dye-conjugated dendrimers at 37 °C.

First, four types of carboxyl-terminal dendrimers with different hydrophobicity (C-den, C-Phe-den, Chex-den, and Chex-Phe-den) were synthesized by reacting with succinic anhydride (Suc), cis-1,2-cyclohexanedicarboxylic anhydride (Chex) and l-phenylalanine benzyl ester (Phe) and the subsequent deprotection, in accordance with our previous report [17]. The bound ratio of Suc, Chex and Phe to these dendrimers were estimated by the ^1^H NMR analyses (Figure S1), which is listed in Table 1. Essentially all terminal groups of these dendrimers were carboxylated and modified with Phe. Then, these dendrimers were labeled with 2–5 green fluorescent dyes. These dendrimers showed similar negative ζ-potentials (Table 1). It is well-known that logP, the octanol-water partition coefficient, is a measure of hydrophobicity. LogP values of the terminal structure in C-den, C-Phe-den, Chex-den, and Chex-Phe-den were calculated as −1.29, −0.27, 0.18, and 1.20, respectively (Table 1) [18], indicating that the terminal structure of Chex-Phe-den was the most hydrophobic among them. The associations of these dendrimers with immune cells were examined, in order to elucidate the relationship between the hydrophobicity of carboxyl-terminal dendrimers, and the association with immune cells, including T cells. The immune cells were collected from mice and incubated with green fluorescent dendrimers for 3 h at 37 °C. Then, these cells were analyzed by flow cytometry. The mean fluorescence intensity of immune cells treated with C-den, C-Phe-den, Chex-den, and Chex-Phe-den were 4.8, 8.6, 7.7, and 95.0, respectively. This indicates that Chex-Phe-den was highly associated with immune cells, but the others were not. T cells and B cells were stained with PE-conjugated anti-CD3 and anti-CD45R antibodies, respectively, and the double-stained cells were examined by the flow cytometry. Chex-Phe-den was bound to both T cells and B cells, and essentially all T cells and B cells were associated with Chex-Phe-den (Figure 2). On the other hand, the other dendrimers were not bound to these cells. The double-positive cells of Chex-Phe-den to T cells and B cells were 20% and 24%, respectively, but those of other dendrimers were less than 4% (Table 1). This indicates that both the Chex group and Phe residue are important in the association with lymphocytes, including T cells. Chex-Phe-den was also incubated with these immune cells at 4 °C, to compare the incubation at 37 °C. The double-positive cells of Chex-Phe-den to both T cells and B cells at 4 °C were much less than those at 37 °C (Table 1). This suggests that Chex-Phe-den was mainly internalized into T cells and B cells via endocytosis.

### 3.2. Biodistribution of the Anionic Dendrimers via Intradermal Administration

**Figure 3:**
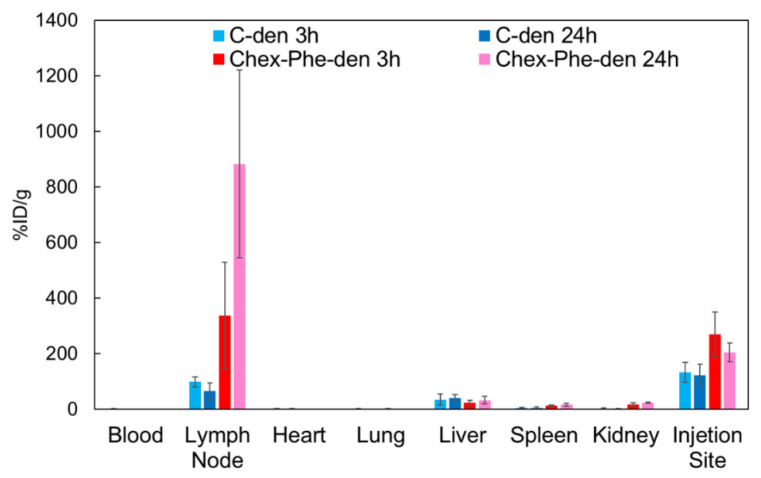
Biodistribution of carboxyl-terminal dendrimers with different hydrophobicity after 3 h and 24 h (*n* = 4).

Chex-Phe-den showed the most efficient internalization into T cells and B cells, so the in vivo studies of Chex-Phe-den were carried out. Our previous work showed the biodistribution study of radiolabeled C-den after the intradermal injection at footpads in mice, indicating that C-den was efficiently accumulated in the popliteal lymph node [10]. In this study, radiolabeled Chex-Phe-den was injected using the same method, and the biodistribution study was performed. Figure 3 showed that Chex-Phe-den was highly accumulated in the lymph node, compared with C-den. The accumulation of Chex-Phe-den in the lymph node tended to increase after 24 h, but C-den tended to decrease. This suggests that the retention of these dendrimers in the lymph node was different. Our results indicate that the biodistribution was affected by the hydrophobicity of the dendrimer.

### 3.3. Fluorescence Imaging of the Lymph Nodes in Mice Injected with Chex-Phe-den

**Figure 4:**
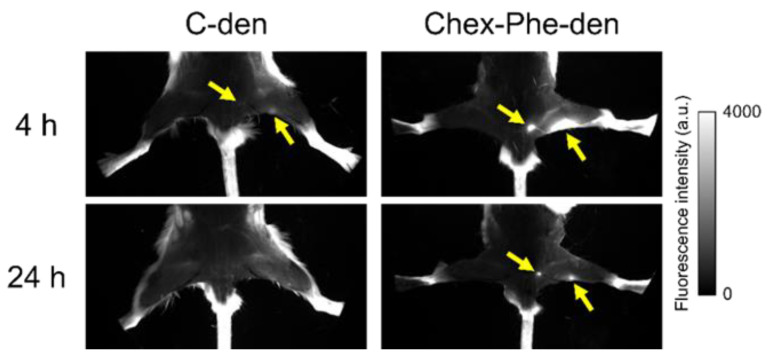
Fluorescence imaging of the lymph nodes after 4 h and 24 h. The arrows indicate the visualized lymph nodes.

Green fluorescent dye-labeled C-den and Chex-Phe-den were intradermally injected into the right rear footpads of the mice, and fluorescence imaging of the lymph nodes was conducted after 4 h and 24 h. These dendrimers could visualize the popliteal and inguinal lymph nodes after 4 h, and the lymph nodes in the Chex-Phe-den-treated mice were brigher than in the C-den-den-treated mice. Chex-Phe-den visualized these two lymph nodes even after 24 h, but C-den did not (Figure 4). This indicates that Chex-Phe-den was highly accumulated in the lymph node and retained there, which is consistent with the biodistribution data (Figure 3).

### 3.4. Association of Chex-Phe-den with Lymph Node-Resident Lymphocytes

**Figure 5:**
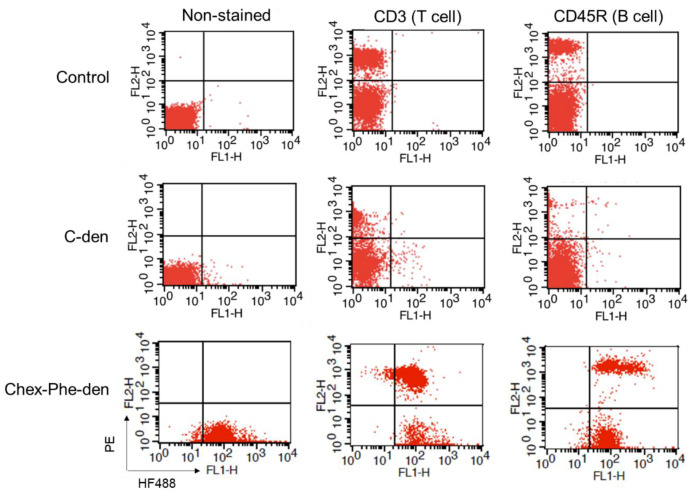
Flow cytometry of PE-stained lymph node-resident lymphocytes in mice injected with the green fluorescent dye-conjugated dendrimers.

The lymph nodes were collected from the mice injected with green fluorescent dye-labeled Chex-Phe-den at 3 h post-injection, and the obtained lymphocytes were analyzed by flow cytometry after the immunostaining. Most T cells and B cells in the lymph node were associated with Chex-Phe-den, but not with C-den (Figure 5). These results indicate that Chex-Phe-den was highly recognized by lymph node-resident T cells and B cells. Thus, the lymphocytes recognition in the lymph node could be controlled by the hydrophobicity in the carboxyl-terminal dendrimer. In other words, the increase in hydrophobicity of dendrimers could increase the association with lymph node-resident lymphocytes, including T cells.

## 4. Discussion

In this study, we examined the association of four carboxyl-terminal dendrimers (C-den, C-Phe-den, Chex-den, and Chex-Phe-den) with immune cells (Figure 1). Chex-Phe-den was associated with lymphocytes (T cells and B cells) at 37 °C, but other dendrimers were not (Figure 2). The size, the surface charge, and the hydrophobicity of the dendrimer are possible factors for enhancing the cell association. Table 1 shows that there are no differences in the ζ-potentials, but the logP values are different. We could not masure their diameters because of the insufficient yields. The diameter may not be largely dependent in the terminal structure without any aggregation, and it is unlikely the dendrimers aggregate each other at the low concentration in the cell association assay. Thus, this suggests that the hydrophobicity of carboxyl-terminal dendrimers is crucial for enhancing the association with immune cells. Chex-Phe-den was not efficiently associated with T cells and B cells at 4 °C (Table 1), so Chex-Phe-den is possibly internalized into these cells via endocytosis. It has been reported that NPs modified with cyclohexyl compounds increased the immune responses, in which the gene expression profile of cytokines linearly increased with the increase in hydrophobicity of the NP [15]. It has been reported that the conjugation of hydrophobic l-phenylalanine ethyl ester to hydrophilic poly(γ-glutamic acid) (γ-PGA) improved the induction level of the antigen-specific cellular and humoral immunities. Additionally, the results showed that interactions of the polymer-based NPs with antigen-presenting cells—dendritic cells and macrophages—could be controlled by changing the type of hydrophobic units, that is, the amino acid grafted to polymers [16]. We reported that attachment of Phe residues to arginine-rich cell-penetrating peptides enhanced the cell internalization through cell membranes [19]. However, these reports did not show any capabilities to deliver into the lymph node-resident lymphocytes.

In this study, Chex-Phe-den was more highly accumulated and retained in the lymph node via the intradermal injection than C-den (Figure 3 and Figure 4). In addition, Chex-Phe-den was highly recognized by the lymph node-resident lymphocytes, including T cells, but C-den was not (Figure 5). This suggests that the hydrophobic effect is largely influenced on the association with immune cells rather than on the flow from the injection site to the lymph node. These results are consistent with our previous report, showing that different anionic dendrimers, such as carboxyl-, sulfonyl- and phosphate-terminal dendrimers, are accumulated in the lymph node, whose association with immune cells are different [10]. In our previous report, the phosphate-terminal dendrimer was associated with B cells, dendritic cells, and macrophages, but not with T cells [10]. Additionally, we previously reported that cationic and nonionic dendrimers showed a much different biodistribution from anionic dendrimers [9]. The report also indicated that anionic dendrimers with G4, G6, and G8, whose diameters were from 6 nm to 14 nm, showed similar biodistribution [9]. Thus, in this range, the dendrimer size is not a main factor to the lymph node-targeted delivery. Our results obtained in this study indicate that the efficiency of the lymph node-targeted delivery is also affected by the hydrophobicity of the dendrimer. The detailed recognition mechanisms by T cells and their immune responses of Chex-Phe-den in T cells remain to be investigated. Additionally, it has been reported that carboxyl-terminal dendrimers modified with Phe showed dual pH- and thermo-sensitivity [17]. Stimuli-responsive delivery systems into the lymph node-resident lymphocytes are possibly developed by using Chex-Phe-den, which is an ongoing project.

## 5. Conclusions

The hydrophobic carboxyl-terminal dendrimer—Chex-Phe-den—was highly accumulated in the lymph node via intradermal injection, and was highly associated with the lymph node-resident lymphocytes. Our results suggest that the hydrophobic effect of carboxyl-terminal dendrimers is crucial for the association and the internalization in lymphocytes. To the best of our knowledge, this is the first report of the lymph node-resident T cells-targeted NPs without any specific T cell ligands. Our findings are of importance to the development of nanoplatforms for cancer immunotherapy.

## Figures and Tables

**Table 1:** Characterization and association with immune cells of different carboxyl-terminal dendrimers.

Dendrimer	Bound Ratio			ζ-Potential (mV)	LogP	Double Positive Cells	
	Suc	Chex	Phe			T Cells	B Cells
**C-den**	~100%	−	−	−21.0 ± 0.9	−1.29	0.74% ± 0.06	1.05% ± 0.10
**C-Phe-den**	~100%	−	~100%	−24.2 ± 1.6	−0.27	1.36% ± 0.08	3.89% ± 0.09
**Chex-den**	−	100%	−	−22.6 ± 2.2	0.18	1.31% ± 0.06	2.20% ± 0.26
**Chex-Phe-den**	−	100%	91%	−19.1 ± 0.2	1.20	20.03% ± 0.57 (4.87% ± 0.35) *	23.81% ± 0.91 (12.09% ± 0.41) *

* 4 °C incubation.

## Supplementary Materials

The following are available online at https://www.mdpi.com/2073-4360/12/7/1474/s1.
