# Supplementary material for: Association of Hydrophobic Carboxyl-Terminal Dendrimers with Lymph Node-Resident Lymphocytes
Source: Polymers (Basel). 2020 Jun 30;12(7):1474. doi: 10.3390/polym12071474 (PMC7408625; doi:10.3390/polym12071474)
Supplement: Supplementary file 1 [file polymers-12-01474-s001.pdf]

Supporting Information

Association of Hydrophobic Carboxyl-Terminal Dendrimers with Lymph Node-Resident Lymphocytes

Yutaka Nishimoto [1], Misaki Nishio [1], Shu Nagashima [1], Kohei Nakajima [2], Takayuki Ohira [2], Shinya Nakai [3], Ikuhiko Nakase [3], Kei Higashikawa [4], Yuji Kuge [4], Akikazu Matsumoto [1], Mikako Ogawa [2] and Chie Kojima [1]*

[1] Department of Applied Chemistry, Graduate School of Engineering, Osaka Prefecture University, 1-1 Gakuen-cho, Naka-ku, Sakai, Osaka 599-8531, Japan

[2] Laboratory of Bioanalysis and Molecular Imaging, Graduate School of Pharmaceutical Sciences, Hokkaido University, Kita-12 Nishi-6, Kita-ku, Sapporo, Hokkaido 060-0812, Japan

[3] Department of Biological Science, Graduate School of Science, Osaka Prefecture University, 1-2, Gakuen-cho, Naka-ku, Sakai, Osaka, 599-8570, Japan

[4] Central Institutes of Isotope Science, Hokkaido University, Kita 15 Nishi 7, Kita-ku, Sapporo, Hokkaido 060-0815, Japan

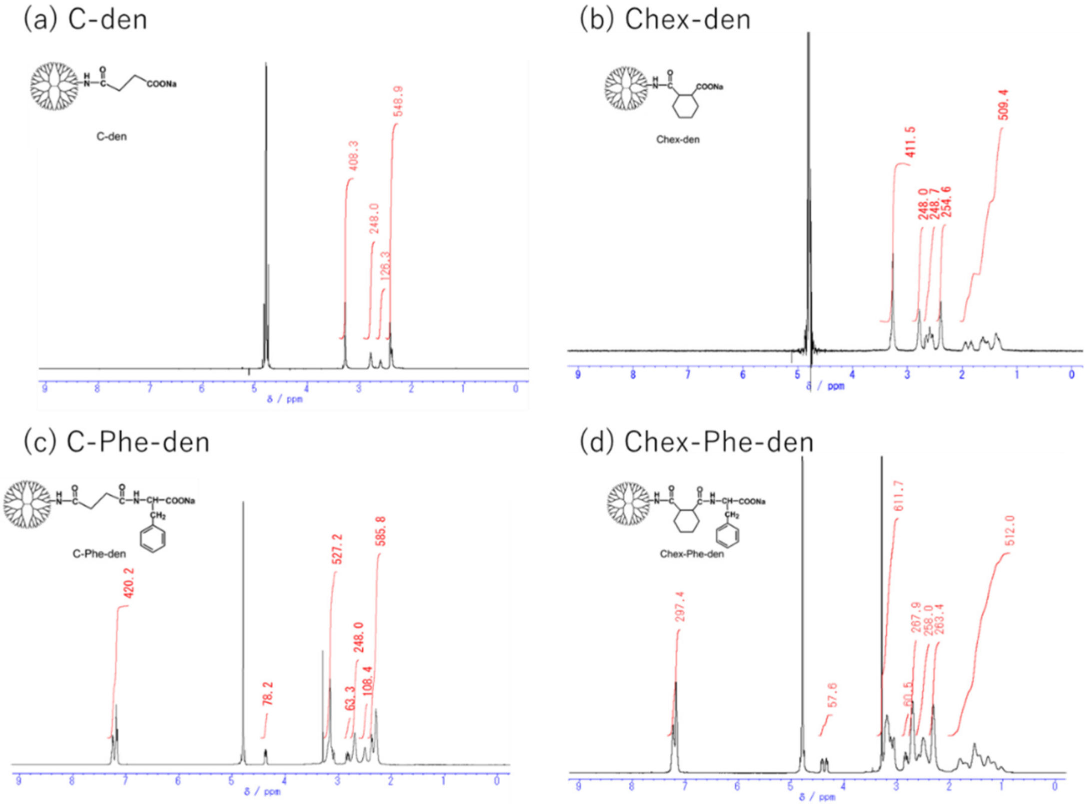

Figure S1. ^{1}H NMR spectra of (a) C-den, (b) Chex-den, (c) C-Phe-den and (d) Chex-Phe-den.
